# Efficacy of endovascular repair in the treatment of retrograde ascending aortic intramural haematoma

**DOI:** 10.1186/s13019-023-02234-0

**Published:** 2023-04-11

**Authors:** Bailang Chen, Rui Zhang, Haibing Liu, Yao Chen, Zanxin Wang, Minxin Wei

**Affiliations:** Department of Cardiac Surgery, The University of Hong Kong-Shenzhen Hospital, Shenzhen, Guangdong People’s Republic of China

**Keywords:** Endovascular repair, Intramural hematoma, Aortic dissection

## Abstract

**Background:**

The current treatment for retrograde ascending aortic intramural hematoma (RAIMH) remains challenging. This study aims to summarize the short-term results of endovascular repair in the treatment of retrograde ascending aortic intramural hematoma.

**Methods:**

Between June 2019 and June 2021, 21 patients (16 males and 5 females) with a retrograde ascending aortic intramural hematoma, aged 53 ± 14years, received an endovascular repair in our hospital. All cases involved an ascending aortic or aortic arch intramural hematoma. 15 patients had an ulcer on the descending aorta combined with an intramural hematoma in the ascending aorta and 6 patients had typical dissection changes on the descending aorta combined with an intramural hematoma in the ascending aorta. All patients had a successful endovascular stent-graft repair, with 10 cases operated on in the acute phase (<14 days) and 11 cases in the chronic phase (14–35 days).

**Results:**

A single-branched aortic stent graft system was implanted in 10 cases, a straight stent in 2 cases, and a fenestrated stent in 9 cases. All surgeries were technically successful. One of the patients developed a new rupture 2 weeks after surgery and was converted to a total arch replacement. No perioperative stroke, paraplegia, stent fracture or displacement, limb or abdominal organ ischemia occurred. The intramural hematomas started being absorbed on CT angiography images before discharge. There was no incidence of postoperative 30-day mortality, and the intramural hematomas in the ascending aorta and aortic arch were fully or partly absorbed.

**Conclusion:**

Endovascular repair of retrograde ascending aortic intramural hematoma was shown to be safe and effective, and correlated with favorable short-term results.

## Background

Acute aortic intramural hematoma (IMH) is defined by the hemorrhage within the aortic wall without intimal tear, which could be developed into an aortic dissection (AD) and/or rupture, resulting in lethal outcomes generally. Retrograde ascending aortic intramural hematoma (RAIMH) is a special type of intramural hematoma that is characterized by the presence of a primary tear or ulcer at the distal end of the aortic arch, with involvement of the ascending aorta and even the aortic arch [1]. The rupture of vasa-vasorum of aorta wall or intimal fracture sourcing from the atherosclerotic plaque progression are currently considered to elicit IMH [2].

The entry tear, PAU, and IMH exhibit a mutual transformation relationship to a certain extent, while the specific mechanism remains to uncover [3–4]. IMH has been suggested to originate from the rupture of vasa vasorum in weak areas of the medial layer of the aortic wall that triggers a tear into the aortic lumen [5–6]. Another speculation is the IMH originating from small intimal tears that leads to thrombosis of these tears makes the tears difficult to detect by imaging [7]. Both could be induced by the elevated wall stresses pressed on the weakened tissue.

Currently, for type A intramural hematomas, ascending aorta and even aortic arch replacement and elephant trunk stent implantation, under deep hypothermic circulatory arrest, are indicated [8]. However, total aortic arch replacement is a complicated surgery that involves prolonged anesthesia and operative time and presents a serious risk of postoperative complications. Compared with open surgery, thoracic endovascular aortic repair (TEVAR) is less invasive. For Stanford type B aortic dissection (TBAD), whether complicated or uncomplicated, TEVAR shows favorable short and mid-term results [9]. As similar to type B aortic dissection and retrograde type A aortic dissection, retrograde type A IMH are also just manifested by an intimal defect (intimal tear or ulcer-like projection) in the descending aorta. Given the similar anatomic of intimal defects in the descending aorta between type B aortic dissection and retrograde type A IMH, few studies have explored the efficacy of TEVAR for retrograde type A IMH, which displayed the considerable outcomes [10–11].

In recent years, we have started to adapt TEVAR to treat retrograde ascending aortic intramural hematomas and have achieved good results. This study is an analysis of these procedures to help determine their efficacy and safety.

## Materials and methods

### General information

We conducted a retrospective study of all consecutive cases of RAIMH patients admitted to the Cardiovascular surgery unit of the University of Hong Kong-Shenzhen Hospital between June 2019 and June 2021.We subcutaneously injected morphine to relive the pain, β-blockers pumped intravenously to control the heart rate, and urapidil veins to reduce blood pressure.The heart rates of all patients were controlled at 60–70 beats/min. The blood pressure of dissection patients should be controlled at systolic blood pressure < 120 mmHg, diastolic blood pressure < 80 mmHg. None of the cases involved connective tissue disease. Before surgery, we selected the appropriate stent by measuring the patient’s aortic arch dimensions. An experienced surgeon (At least 10 years of interventional surgery experience, not less than 1000 interventional operations) used preoperative computed tomography angiography (CTA) to determine the diameter of the left subclavian artery (LSA) lumen, the distance between the LSA and left common carotid artery (LCCA), the distance between the LSA and the primary tear, and the aortic diameters of the proximal and distal landing zones.

**Fig. 1:**
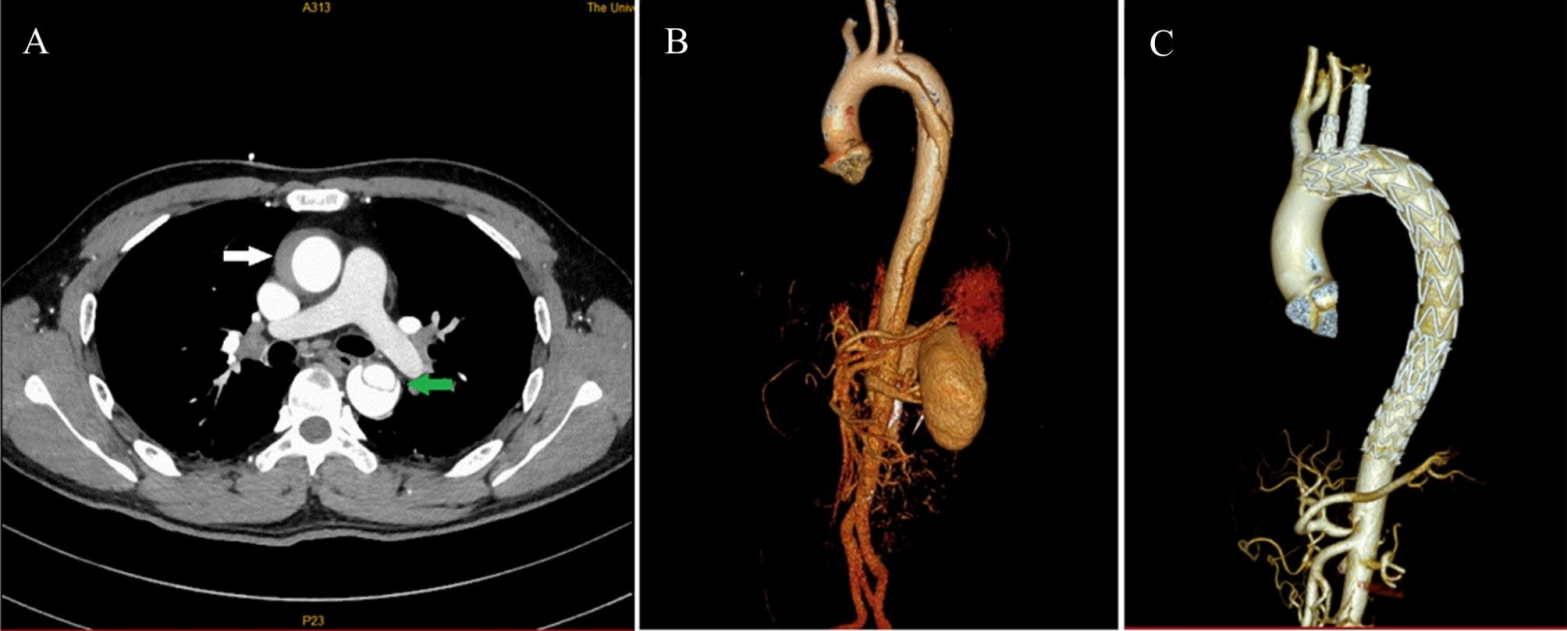
**A** Preoperative CT scan showing the ascending aortic intramural hematoma (white arrow indicating abnormal soft tissue in the wall of the aorta) and acute type B aortic dissection involving the descending thoracic aorta(green arrow indicating intimal in descending aorta). **B** Preoperative three-dimensional aortic IMH. **C** Postoperative three-dimensional imaging of the aorta

Inclusion Criteria: (1) diagnosed with RAIMH based on CTA; (2) no uncontrolled hypertension or intractable pain. Exclusion criteria: (1) previous thoracic aortic replacement or TEVAR; (2) conservative treatment or abandoned treatment; (3) the primary tears or ulcers were located at the ascending aorta or the aortic arch (10 patients were excluded). During the study review period of June 2019 to June 2021, we found that 10 patients with RAIMH were treated by endovascular repair with the single-branched stent or grafts, 2 by endovascular repair with a straight stent, and 9 by a fenestrated stent. All patient comorbidities are listed in Table 1. Table 2 reports the characteristics of the 21 included patients. There were 16 men and 5 women with a mean age of 53 ± 14 years (range 32–82 years). All were diagnosed by high-resolution CT scans (Fig. 1A). A 64-row CT scanner (LightSpeed VCT, GE Healthcare, Japan) was utilized on all patients for examination. The 1.0-mm CT arterial phase thin-slice image data were collected for CTA reconstruction and analysis. Iodine, non-ionic contrast agent—iomeprol (Iomeron 400, Bracco UK Ltd, Great Britain)—was intravenously administered to the veins of the cubital fossa through the automatic syringe. Contrast agent of 50 ml was intravenously administrated with the infusion rate of 5.0 ml/s. CTA data were transferred to a computer workstation (AW 4.7, Healthcare) for analysis. 3D reconstructions of the aorta and branch vessels showed that in all cases the primary tears were located at the distal end of the aortic arch or the descending aorta but the lesion involved the ascending aorta and even the aortic arch(Fig. 1B). All patients underwent TEVAR using single-branched stent grafts, straight stent graft or fenestrated stent according to the location of primary intimal tear. (Fig. 1C).

**Table 1:** Characteristics of the 21 patients treated by TEVAR for retrograde ascending aortic intramural hematoma with a primary intimal tear or ulcer like projection in the descending thoracic aorta

Variables	Patients (n = 21)
Mean age (SD) -years	53 ± 14
Sex-male [n(%)]	16(76)
Sex-female [n(%)]	5(24)
Smoking history [n(%)]	4(19)
Symptom [n(%)]	
Chest and back pain	17(81)
Abdominal pain	3(14)
Hemiplegia	1(5)
Comorbidities [n(%)]	
Diabetes mellitus	1(5)
Dyslipidaemia	5(24)
Hypertension	15(71)
Coronary artery disease	1(5)
Prior TIA/CVI	0(0)
Atrial fifibrillation	0(0)
Chronic renal disease	1(5)
Chronic obstructive pulmonary disease	1(5)
Peripheral arterial disease	1(5)
Laboratory findings [n(%)]	
High uric acid	4(19)
Pericardial effusion	1(5)
D-dimer>1	21(100)
LVEF(%)	65.2 ± 5.9
Extent of dissection/IMH in descending aorta[n(%)]	
Above the diaphragm	3(14)
Below the diaphragm	18(86)

TIA = transient ischaemic attack; CVI = cerebral vascular insuffificiency; LVEF = left ventricular ejection fraction.

**Table 2:** Contrast-enhanced computed tomography angiogram parameters and intervention details in patients with RAIMH

No.	Age(year)/Sex	Operation time(min)	Anesthesia	DIT-LSA-mm	TEVAR timing (days)	Lesion in DTA
1	32/M	60	GA	10	14	IMH
2	45/M	180	GA	20	21	dissection
3	36/M	90	GA	15	15	dissection
4	59/M	61	GA	16	10	dissection
5	66/F	190	GA	21	14	IMH
6	49/M	61	GA	15	14	IMH
7	43/M	65	GA	7	35	dissection
8	54/F	50	GA	15	16	IMH
9	46/M	64	GA	16	14	IMH
10	58/M	60	GA	10	15	dissection
11	36/M	35	GA	11	10	IMH
12	68/F	44	GA	20	30	IMH
13	56/M	69	GA	8	8	IMH
14	48/M	45	GA	21	1	IMH
15	43/M	60	GA	10	1	IMH
16	82/F	81	GA	0	8	IMH
17	35/M	70	GA	10	6	IMH
18	47/M	56	GA	15	14	IMH
19	56/M	42	GA	5	7	dissection
20	72/F	60	GA	8	12	IMH
21	78/M	80	GA	5	10	IMH

IMH = intramural hematoma; DIT-LSA = distance between the primary intimal tear and the left subclavian artery;

TEVAR timing = time from the initial presentation until surgery. DTA = descending thoracic aorta; GA = General anesthesia.

### Definition

PAU is defined as an aortic atherosclerotic lesion in the internal elastic lamina penetrating the media generally accompanied by aortic calcification plaque [12]. RAIMH was defined as TAIMH with PAU or entry tear in the descending aorta. The CTA parameters refers to the maximal ascending aortic diameter (MAAD), the maximal ascending aortic hematoma thickness (MAAHT), the descending aortic diameter (DAD), and the FL diameter or IMH thickness, which were measured at near pulmonary artery bifurcation level.

### Operation details

**Fig. 2:**
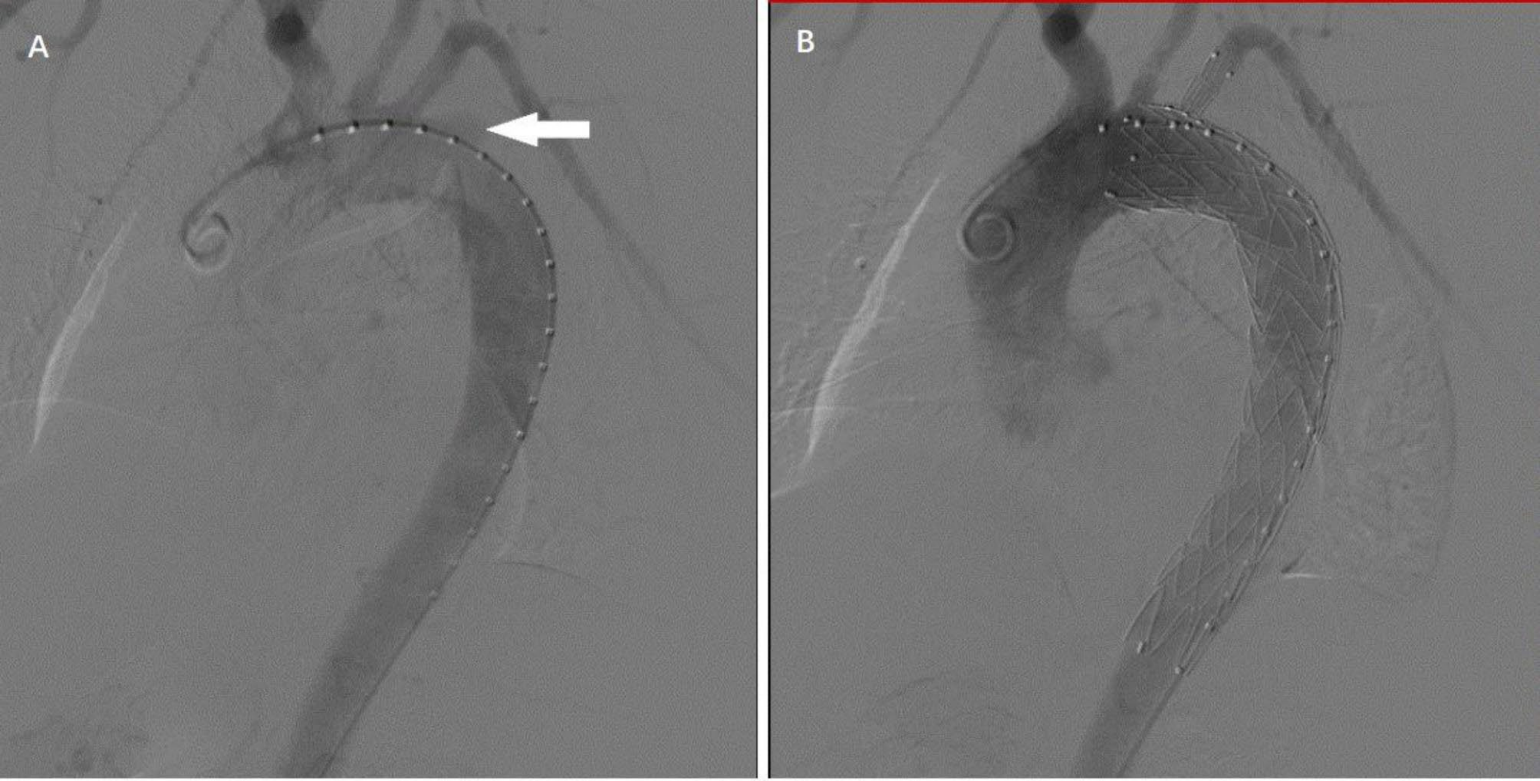
**A** Aortic angiography suggests that the primary tear involved the LSA. **B** After the release of the stent, the three branches were unobstructed, the primary tear was closed, and no endoleak was found

The implanted thoracic stent grafts were Castor single-branched stent grafts (Microport Medical, Shanghai, China) and Relay (Bolton Medical, Sunrise, FL, USA). The operation was performed in a digital subtraction angiography (DSA) operating room under general anesthesia. The surgical method was as follows: The right femoral artery was cannulated, and the guide wire and pigtail catheter were advanced into the ascending aorta under fluoroscopic guidance. Then, an aortogram was performed to identify the proximal primary tear and to measure the diameter of the relevant segments of the aorta to ensure that the correctly sized device had been prepared (Fig. 2A). TEVAR was aimed at excluding the penetrating aortic ulcer (PAU) or entry tear in the descending aorta. When the proximal landing zone affected the blood flow of the aortic arch branch, one or combined adjunctive procedures were employed, including handmade pre-fenestrated stent grafts. The handmade pre-fenestrated stent graft was made according to the preoperative CTA and the intraoperative aortogram findings, through which we marked the stent by measuring the distance among the aortic arch branch vessels. To achieve an adequate landing, single-branched stent grafts were performed in some patients, as required. The stent graft was oversized relative to the mean aortic diameter of the proximal landing zone by 5–10%. Blood pressure was controlled throughout the procedure to maintain a systolic pressure of 70–90 mmHg. After the stent was released, aortography was immediately performed to evaluate the patency of the side branch and to examine whether endoleak occurred and whether the entry tear was sealed (Fig. 2B).

### Stent graft selection

Generally, fenestrated stents or single-branch stents were selected for patients with the distance between the primary intimal tear and the LSA < 15 mm. If the primary intimal tear was found located on the greater curvature of the aortic arch, the single-branch stents were preferentially selected to completely cover the lesion. For patients with primary intimal tear on the minor curved side of the aortic arch, the Relay stent was preferentially selected, which can reduce the occurrence of “beak” on the minor curved side with its better flexibility. For partial retrograde dissection patients, single-branch or fenestrated stents will be employed to coverage retrograde dissection intimal or thick hematoma that near LSA despite the distance between the primary intimal tear and the LSA > 15 mm. The lateral branch of the single-branch stent moved back 5 or 10 mm lied the common specification. The intimal tear of some patients’ primary was located in LSA, the fenestrated stent would be chosen to reach the anchoring area to 15 mm or above as far as possible, considering the insufficient anchoring length of the single-branch stent was. For some patients, the the LSA is close to the LCCA, the side branch will be put into the LCCA and fenestrated behind the branch to avoid the LSA, as the application of a single branch stent will block the LCCA (Table 3).

**Table 3:** Stent choice in patients with retrograde ascending intramural haematoma (IMH)

No.	Proximal landing zone	Number of stent grafts	Procedure
1	2	1	Single branch stent
2	1	2	Handmade fenestrated behind single branch stent + LSA stent
3	2	1	Single branch stent
4	2	1	Single branch stent
5	1	2	Handmade fenestrated before single branch stent + LCCA stent
6	3	1	Relay stent
7	2	1	Single branch stent
8	3	1	Relay stent
9	2	1	Single branch stent
10	2	1	Fenestrated
11	2	1	Fenestrated
12	2	1	Fenestrated
13	2	1	Single branch stent
14	2	1	Fenestrated
15	2	1	Single branch stent
16	2	1	Fenestrated
17	2	1	Fenestrated
18	2	1	Fenestrated
19	2	1	Single branch stent
20	2	1	Fenestrated
21	2	1	Fenestrated

### Clinical follow-up

**Fig. 3:**
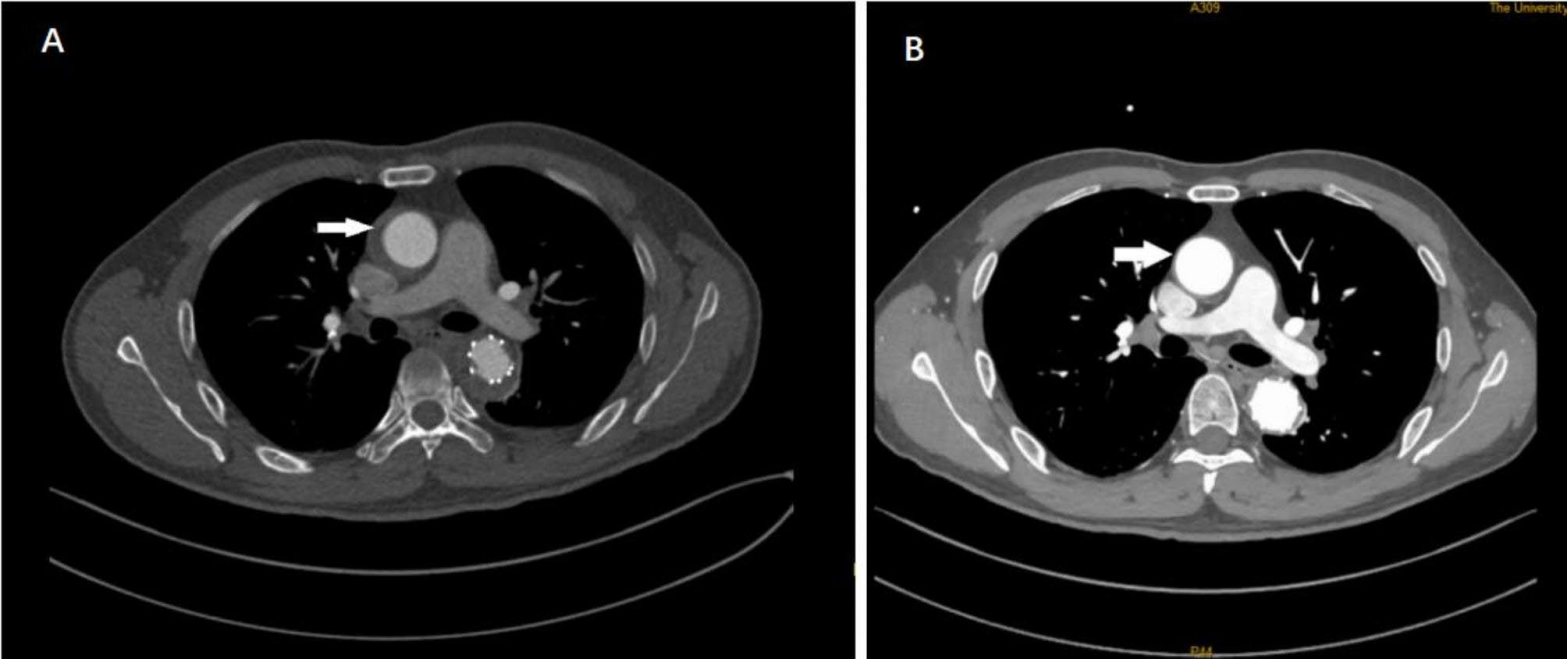
**A** Follow-up CT scan, 2 weeks postoperatively with resolution of type A IMH and satisfactory positioning of the TEVAR. Image showing that the ascending aortic hematoma is smaller than before. **B** Follow-up CT scan, 3 months postoperatively with resolution of the type A IMH. Image showing ascending aortic hematoma were completely absorbed

Patients were followed up by telephone interview or through the outpatient clinic. The clinical follow-up referred to a physical examination, routine biochemical and profile (duration of follow-up and, if the patient died, the cause of death). Patients with hypertension were monitored and systolic blood pressure was maintained between 100 and 120mmHg by antihypertensive drugs. After the operation, aspirin (100 mg qd) was given routinely for three to six months. For all patients, the follow-up protocol included assessment of clinical symptoms, survival, and CTA images at 1, 3, 6, and 12 months and then annually (Fig. 3A,B). Follow-up data were obtained by reviewing medical records and a telephone interview. The side branch patency, false lumen, thrombosis, endoleak events, and patient survival were recorded.

### Statistical analysis

The data were analyzed with SPSS version 17.0 (Chicago) and GraphPad Prism 9.3. Continuous variables are presented as mean ± standard deviation, and categorical variables are shown as proportions. Between-group comparisons of continuous variables were compared using two-tailed t-tests. A p-value < 0.05 was considered to indicate statistical significance.

## Results

### Perioperative results

An immediate postoperative aortogram demonstrated successful exclusion of the lesion and good perfusion of the LSA in all cases. Implantation of the stent was successful in all cases and the lesion was completely sealed. Intraoperative radiography showed that the stent was unobstructed and no endoleak occurred. The technical success rate of the TEVAR was 100%. No patients have developed renal events with dialysis demand, intracerebral haemorrhage and cerebral infarction, all ambulating well. No significant transformation was observed in left upper extremity blood pressure before and after operation. Patient No.2 had an LCCA branch stent during the operation. An 0.5 × 0.5cm^2^ hole was fenestrated behind the branch to prevent the LSA from being blocked and a 10 × 40 mm stent graft was placed in the LSA through the opening. Patient No.5 received a single-branch stent during the operation. A 1 × 1cm^2^ hole was fenestrated before the branch to prevent the LCCA from being blocked and a 9 × 40 mm stent graft was placed in the LCCA through the opening. Intraoperative radiography showed that the stent was unobstructed and no endoleak occurred.

### Follow-up results

**Fig. 4:**
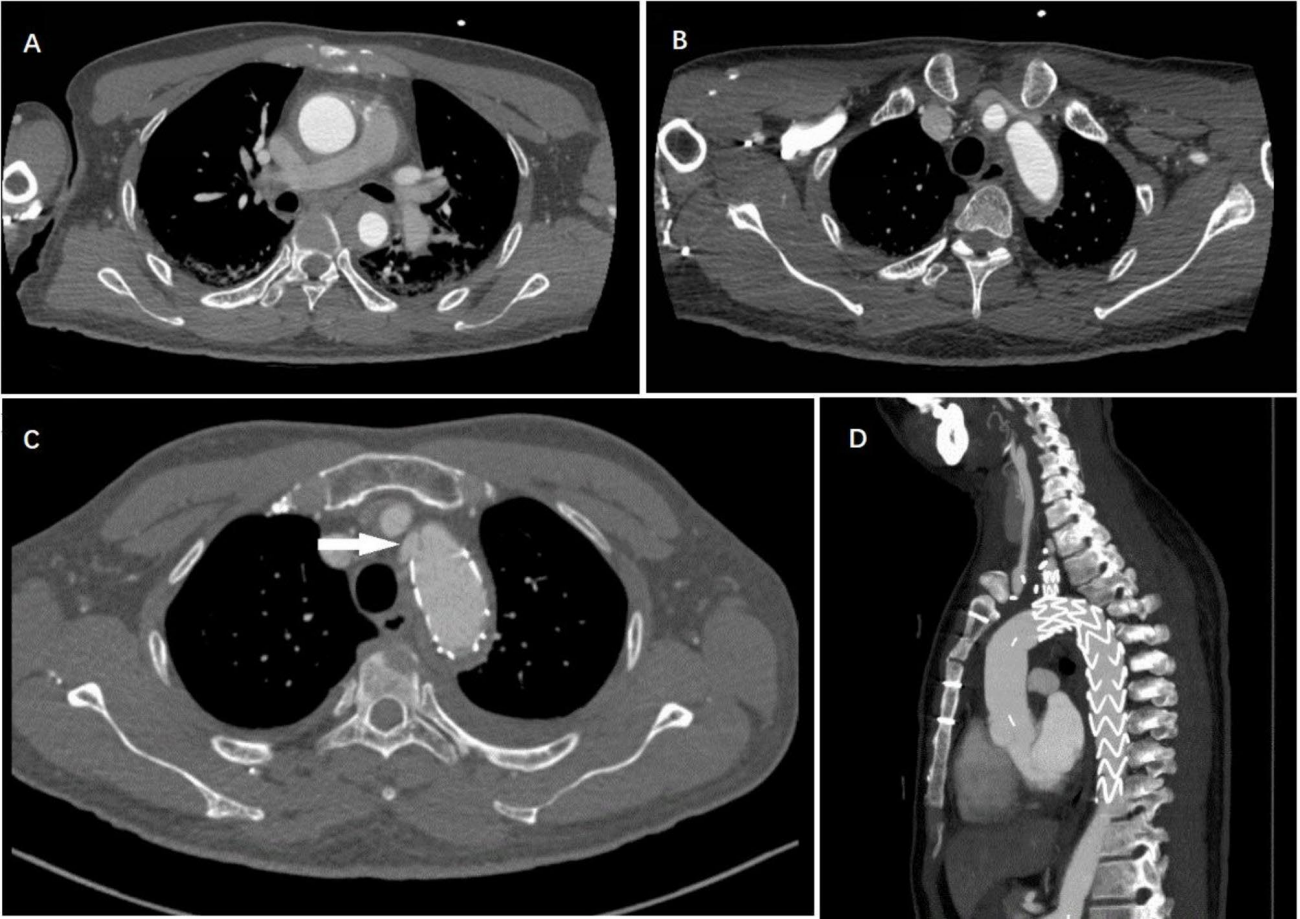
**A,B** A patient with a Type A intramural hematoma received TEVAR treatment on the first day of onset, and the chest pain persisted after the operation. **C** 2 weeks after TEVAR, a new CT scan showed a new rupture at the proximal end of the stent (white arrow). **D** We performed total arch replacement surgery

**Fig. 5:**
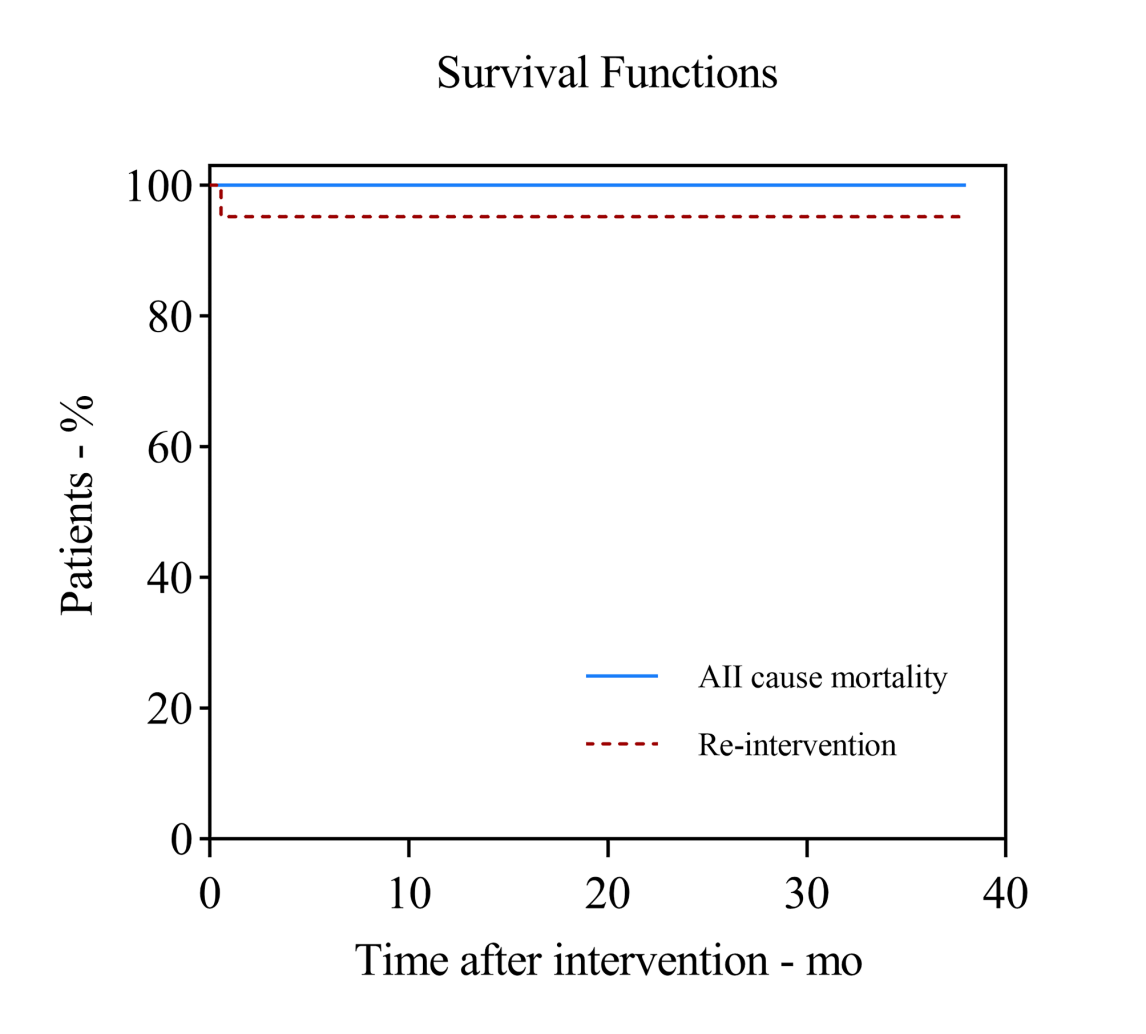
Cumulative Kaplan-Meier estimate for freedom from all-cause mortality (solid blue line) and re-intervention (dashed red line) in patients treated by thoracic endovascular repair for retrograde type A intramural haematoma

After surgery, patients were monitored for procedure-related complications for 12–38 months. Follow-up included whether the patient had died, the cause of death, complications, current symptoms, and the blood pressure in both upper extremities. CT scans were acquired at 1, 6, and 12 months, then annually. The side branch patency, false lumen thrombosis, endoleak events, and patient survival were recorded. All patients were followed up regularly after surgery. There were no unusual discomfort and no cerebral or left upper limb ischemia during the follow-up period. Hypertensive patients received effective blood pressure control, and the whole cohort did not have any perfusion or blood pressure issues. Patient No. 15 developed persistent chest pain after the operation. During the follow-up, a new rupture at the proximal end of the stent was found in patient No. 15, which may result from the emergency operation of the patient, that the relatively fragile aortic wall in acute phase may fail to tolerate the deployed stent grafts (Fig. 4). The emergency surgery was carried out considering the pericardial effusion, pleural effusion, a transient decrease in blood pressure indicated in the patient, and we were afraid at risk of aortic rupture. During a mean follow-up of 22 ± 8months (12–38months), there was no recurrence of symptoms, no endoleak was noted in any of the patients, and the latest CTA images showed that all the IMH in the ascending aorta and the false lumen at the stent level were completely or nearly absorbed (Fig. 5).

### Aortic remodeling

**Fig. 6:**
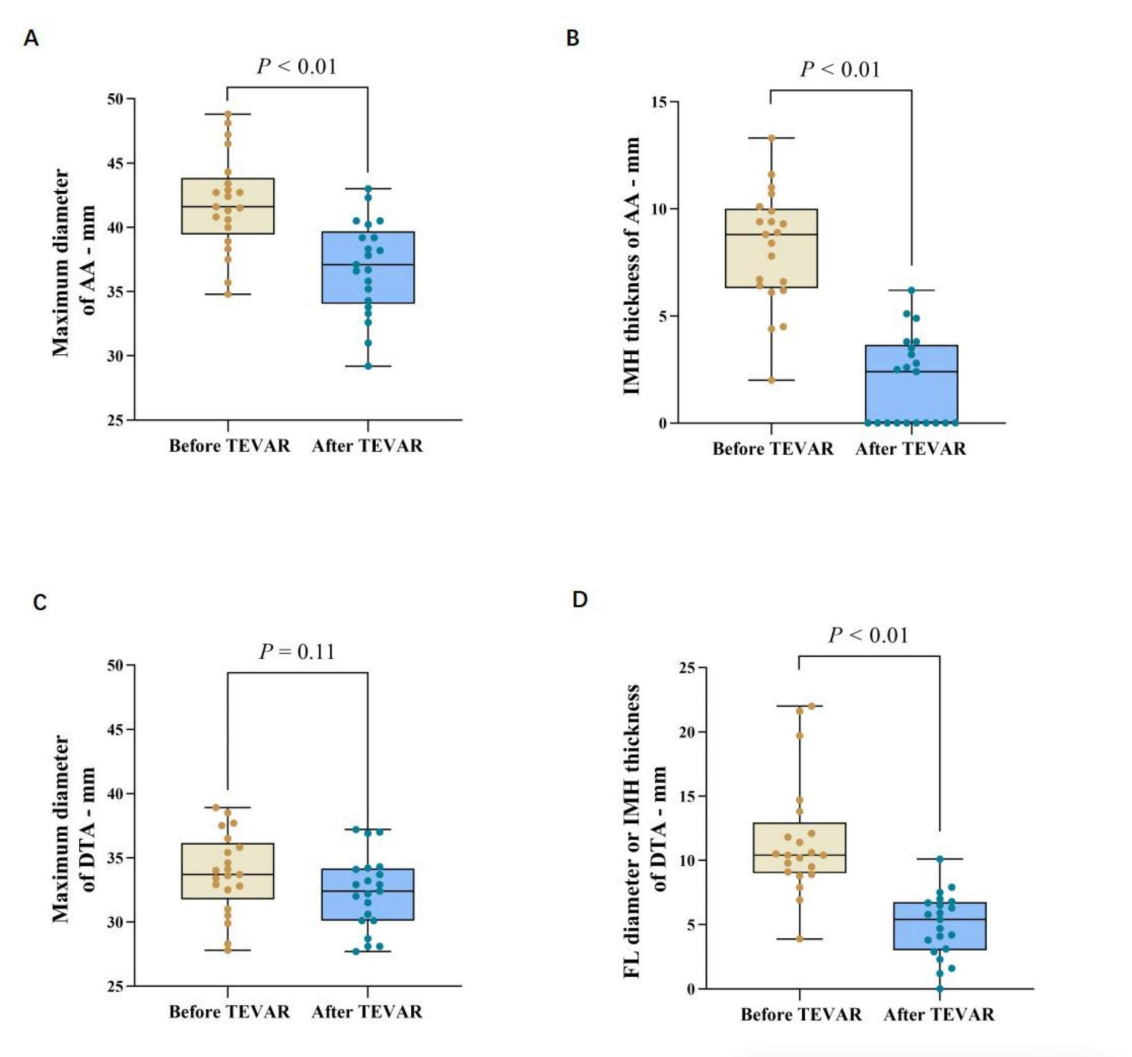
Box plot shows the changes of different parameters before and after surgery. **A, B** Measurement with computed tomography (CT) after TEVAR showed that the maximum diameter, and the intramural haematoma (IMH) thickness of the ascending aorta (AA) decreased significantly from measurements obtained before TEVAR. **C,D** At the level of the pulmonary artery bifurcation, the maximum diameter, and the false lumen (FL) diameter or IMH thickness of the descending aorta (DTA) were significantly reduced from the CT measurements before TEVAR.

The mean maximal ascending aortic diameter (MAAD) and the mean maximal ascending aortic hematoma thickness (MAAHT) were 41.9 ± 3.8 and 8.2 ± 2.7 mm, respectively. The maximum diameters of the descending thoracic aorta (DTA) and FL diameter or IMH thickness at the pulmonary bifurcation level were 33.8 ± 3.1 and 11.6 ± 4.6 mm, respectively. Complete IMH resolution in the ascending aorta (AA) was observed on the first post-operative follow-up CTA in all patients. The AA and DTA diameters were significantly decreased at the last follow-up CTA after TEVAR. The mean maximum diameter and IMH thickness of the AA decreased to 36.9 ± 3.6 mm (p < 0.01) and 1.9 ± 2.1 mm, respectively, after TEVAR (p < 0.01). At the pulmonary artery bifurcation level of the stent graft in the DTA, the mean diameter of DTA decreased to 32.3 ± 2.9 mm (p = 0.11) and FL diameter or IMH thickness to 4.9 ± 2.5 mm after TEVAR (p < 0.01) (Table 4) (Fig. 6).

**Table 4:** Aortic diameters of the 21 patients before and after TEVAR for retrograde ascending aortic intramural hematoma (IMH) with a primary intimal tear or ulcer-like projection in the descending thoracic aorta

Diameters-mm	Pre-TEVAR (n = 21)	Post-TEVAR (n = 21)	Diameter difference	p
Ascending aorta				
Aortic diameter	41.9 ± 3.8	36.9 ± 3.6	5.0 ± 2.6	< 0.01
IMH thickness	8.2 ± 2.7	1.9 ± 2.1	6.2 ± 2.3	< 0.01
Descending aorta				
Aortic diameter	33.8 ± 3.1	32.3 ± 2.9	1.5 ± 2.3	0.11
FL diameter or IMH thickness	11.6 ± 4.6	4.9 ± 2.5	6.7 ± 4.7	< 0.01

FL = false lumen.

## Discussion

Intramural haematoma (IMH) of the ascending aorta is an occasional variant of the acute aortic syndrome, which is a life-threatening condition when left untreated [13]. In the European Society of Cardiology (ESC) guidelines the definition of type A IMH is a circular or crescent-shaped thickening of > 5 mm of the aortic wall with the absence of a dissecting membrane, intimal disruption, or false lumen flow, involving the ascending aorta and aortic arch [14]. The incidence of IMH in aortic dissection patients varies between 10% and 30% in the literature, and IMH more frequently involves the descending aorta (58%) rather than the arch or ascending aorta (42%) [15]. Retrograde type A IMH is a special subtype of IMH with the primary intimal disruption located in the descending aorta. In recent studies, this has been termed retrograde RAIMH [16]. There is still a lack of clear and sufficient evidence to support optimal surgical management. In this study, we reported the feasibility and early promising results of utilizing a stent graft in the treatment of RAIMH with the primary entry tear at the far end of the aortic arch.

At present, reports on the condition of retrograde type A IMH are rare, and management of this condition still lacks consensus [10]. Type A IMH has a higher risk of neurological or cardiac complications and mortality. Some researchers believe that hybrid treatment with ascending aortic replacement and distal thoracic aortic endovascular repair (TEVAR) or using the frozen elephant trunk procedure is the most appropriate treatment for acute retro-Type A IMH [17–18]. Although traditional surgical repair is thought to be an effective therapy for type A IMH, it is reported to be associated with significant morbidity and mortality and is not suitable for frail patients [19]. TEVAR is a valid alternative for patients with prohibitive surgical risk, although the landing zones can be unsuitable and the risk of neurologic and cardiac complications can be high [20–21]. Many concerns remain about TEVAR utilization in the treatment of such diseases. The main concerns surround the potential that the stent will cause iatrogenic damage, leading to aortic rupture or the appearance of a new aortic dissection, and the possibility that the part of the ascending aortic hematoma that is not covered by the stent will continue to progress [15]. In this study, all patients underwent TEVAR, which aims to exclude the primary intimal tear in the descending aorta; 20 of the 21 patients showed clinically good outcomes. The patients included in our study had good outcomes. The reasons for these good outcomes may stem from patient selection, namely the strict control of the patient’s blood pressure (100–120/60-80mmHg) and heart rate (60–70/min), the exclusion of entry tears in the ascending aorta, exclusion of intractable pain and refractory hypertension, surgery timing was assisted by echocardiographic imaging of the changes of the ascending aortic hematoma, and not using stents with a proximal bare-spring configuration.

At present, the use of TEVAR to treat this type of intermural hematoma has rarely been reported and there is little research on the treatment of the arch in RAIMH. Song et al. [10] reported 10 patients with RAIMH who underwent TEVAR, which aims to exclude the primary intimal tear in the descending aorta, and all included patients had good clinical outcomes; the technical success rate of TEVAR was 100%. In his study, two patients underwent urgent TEVAR due to persistent pain, and the remaining eight patients underwent delayed TEVAR. During a mean follow-up of 29.8 months (6–46 months), the CTA images showed that all the IMHs in the ascending aorta and the false lumens at the stent level were completely absorbed. Haenen et al. [11] reported two patients who underwent TEVAR for acute TBAD with type A IMH and also showed good outcomes. Two patients received emergency treatment by endovascular means instead of open surgery, with satisfactory short-term and one-year follow-up results. Thus, TEVAR may be an acceptable treatment option for patients in this condition.

In our study, 21 patients underwent successful surgery and stents were successfully released. No stent displacement or endoleak was identified during intraoperative angiography. After follow-up, it was found that the hematoma of the ascending aorta was almost completely absorbed and the branches of blood vessels remained unobstructed. Perioperative mortality was 0. During the follow-up period, there were no paraplegia, cerebral infarction, or left upper extremity ischemia. We identified a new rupture at the proximal end of the stent in one patient, two weeks after surgery. We performed artificial blood vessel replacement surgery on this patient.

However, caution must be taken, during the endovascular procedure, when deploying the device in the aorta, to not escalate the IMH into a true type A aortic dissection. Correct sizing of the prosthesis is important to prevent such escalation. When performing TEVAR, choosing appropriate stents is very important to prevent the IMH from escalating into AD. There is consensus that the ideal proximal seal zone for TEVAR should be in a healthy segment of the aorta. As some investigators have shown, landing on the IMH may increase the risk of retrograde type a aortic dissection (RAAD) after TEVAR [22]. In the current series the rate of RAAD was 4%, and another study also reported the occurrence of RAAD after TEVAR in retrograde type A IMH [23]. There are several possible mechanisms. Firstly, as the stent grafts are deployed on the aortic segment with IMH, the intima may be injured, especially when there is excessive oversizing. Secondly, the manipulation of wires and catheters during TEVAR may cause damage to the intima of the ascending aorta. Thirdly, in the IMH of the ascending aorta, there may be tiny and unrecognizable intimal tears that can gradually enlarge and lead to the development of RAAD [24]. In our study, we opted to utilize 5–10% oversizing and found that only one dissection occurred in our cohort. Retrograde type A aortic dissection (TAAD) after TEVAR for TBAD is a recognized potentially lethal complication with an incidence of 1.3–11%. Risk factors for retrograde TAAD after TEVAR include stent graft oversizing, use of a proximal bare-spring stent graft, aortic arch dilatation, a proximal tear site within the arch, notable “bird’s beaking and stent graft landing proximal to the LSA [25–27]. Risk factors for adverse aortic events in previous studies include maximal hematoma thickness (MTH) > 11 mm, aortic maximum aortic diameter (MAD) ≥ 50 mm, focal intimal rupture, etc. Patients in our study with a hematoma thickness of >10 mm did not have adverse aortic events. To further increase the hematoma coverage area, we choose a single-branch stent or fenestrated in single-branch stent, as appropriate, to expand the hematoma contact area and promote the absorption of the hematoma.

The optimal timing for TEVAR applied in retrograde type A IMH remains a controversial issue and the timing of endovascular treatment is very important for patient prognosis and survival. Several studies have demonstrated that initial timely medical management can achieve good outcomes for type A IMH [28–30]. Some investigators have suggested that appropriate delays in instituting interventions can lower the risk of complications of TEVAR in stable patients [31]. The reasons for this are as follows: firstly, the aortic wall fragile in the acute phase and not able to tolerate the deployed stent grafts; secondly, it’s important to ensure there is no newly developed focal intimal disruption or significantly progressed hematoma in the ascending aorta, which may indicate the need for open surgery [24]. Inflammation and edema occur in the arterial wall and intimal tear in the acute stage of AD, at this time the aortic wall is relatively fragile and vulnerable to damage thus making it prone to serious iatrogenic complications. Based on our experience, we made great efforts to stabilize blood pressure and control chest pain; after around 2 weeks, we repeated the angio-CT to evaluate the evolution of the hematoma, considering the increase in extension, diameter, and appearance of the penetrating ulcer of the aortic wall (PAU). TEVAR was determined if the patient still met the inclusion criteria.This approach allowed the positioning of a thoracic endoprosthesis to completely cover that part of the thoracic aorta where the hematoma was located. Not only that, the size of the ascending aortic hematoma was assessed by bedside ultrasound; progression was an indication for a prompt CTA. If a patient exhibits a risk of a ruptured aorta (pericardial effusion, pleural effusion, hypotension), emergency surgery will be scheduled.

**Fig. 7:**
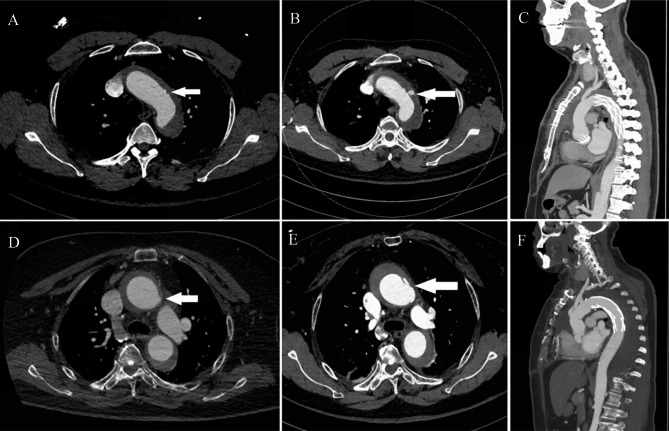
**A,B** A patient with calcification plaque (white arrow) at admission and 15 days after conservative treatment, with a new intimal rupture in the aortic arch near the calcified area. **C** We performed total arch replacement surgery for the patient. **D,E** Another patient with penetrating atherosclerotic ulcer (PAU, 7 × 2 mm, white arrow) at admission and 8 days after conservative treatment; showed a new intimal rupture in the original ulcer area. **F** We performed total arch replacement surgery for the patient

In addition to these 21 patients, two individuals with intramural hematoma of the ascending aorta formed a new PAU on the aortic arch that could not be protected by the stent during the waiting period and a thoracotomy was performed (Fig. 7). Ulceration and calcification may be risk factors for the progression of a type A hematoma. One of our patients underwent TEVAR surgery on the first day of onset; a re-examination of the aortic CTA revealed a new break near the stent after 2 weeks. Thus, the timing of surgery is critical and a conservative approach may not be suitable for such patients. However, the optimal time for TEVAR maybe 7-14days because the aortic wall in acute phase may be fragile to tolerate the deployed stent grafts and we need to make sure there is no newly developed focal intimal disruption or progressed hematoma in ascending aorta,which may otherwise indicate for open surgery.

## Conclusion

We showed that covering the entry tear by TEVAR might be an acceptable solution in selected patients with retrograde ascending aortic intramural hematoma. This less invasive surgical option may play an important future role in a subset of patients with a RAIMH and need to be considered in their treatment strategy. Nevertheless, it is of great significance to evaluate the patient suitability, appropriate time for operation, and surgical techniques for the successful TEVAR.

## Data Availability

The datasets used and/or analysed during the current study are available from the corresponding author on reasonable request.
